# Ten quick tips for sequence-based prediction of protein properties using machine learning

**DOI:** 10.1371/journal.pcbi.1010669

**Published:** 2022-12-01

**Authors:** Qingzhen Hou, Katharina Waury, Dea Gogishvili, K. Anton Feenstra

**Affiliations:** 1 Department of Biostatistics, School of Public Health, Cheeloo College of Medicine, Shandong University, Shandong, P. R. China; 2 National Institute of Health Data Science of China, Shandong University, Shandong, P. R. China; 3 Department of Computer Science, Bioinformatics Group, Vrije Universiteit Amsterdam, Amsterdam, the Netherlands; SIB Swiss Institute of Bioinformatics, SWITZERLAND

## Abstract

The ubiquitous availability of genome sequencing data explains the popularity of machine learning-based methods for the prediction of protein properties from their amino acid sequences. Over the years, while revising our own work, reading submitted manuscripts as well as published papers, we have noticed several recurring issues, which make some reported findings hard to understand and replicate. We suspect this may be due to biologists being unfamiliar with machine learning methodology, or conversely, machine learning experts may miss some of the knowledge needed to correctly apply their methods to proteins. Here, we aim to bridge this gap for developers of such methods. The most striking issues are linked to a lack of clarity: how were annotations of interest obtained; which benchmark metrics were used; how are positives and negatives defined. Others relate to a lack of rigor: If you sneak in structural information, your method is not sequence-based; if you compare your own model to “state-of-the-art,” take the best methods; if you want to conclude that some method is better than another, obtain a significance estimate to support this claim. These, and other issues, we will cover in detail. These points may have seemed obvious to the authors during writing; however, they are not always clear-cut to the readers. We also expect many of these tips to hold for other machine learning-based applications in biology. Therefore, many computational biologists who develop methods in this particular subject will benefit from a concise overview of what to avoid and what to do instead.

## Introduction

Machine learning and deep learning have become the mainstay of bioinformatics analyses and prediction methods [1–4]. Some excellent overviews are available that provide a comprehensive introduction into machine learning approaches within biology [3] or explicitly aim to improve best practices [2,4–9]. For those new to developing machine learning applications within biology, we recommend first reading Greener and colleagues [3] who give an excellent and accessible overview of general concepts, different types of machine learning problems, and discuss a variety of often used methods and architectures, as we do not introduce these topics in depth in this work.

**Fig 1:**
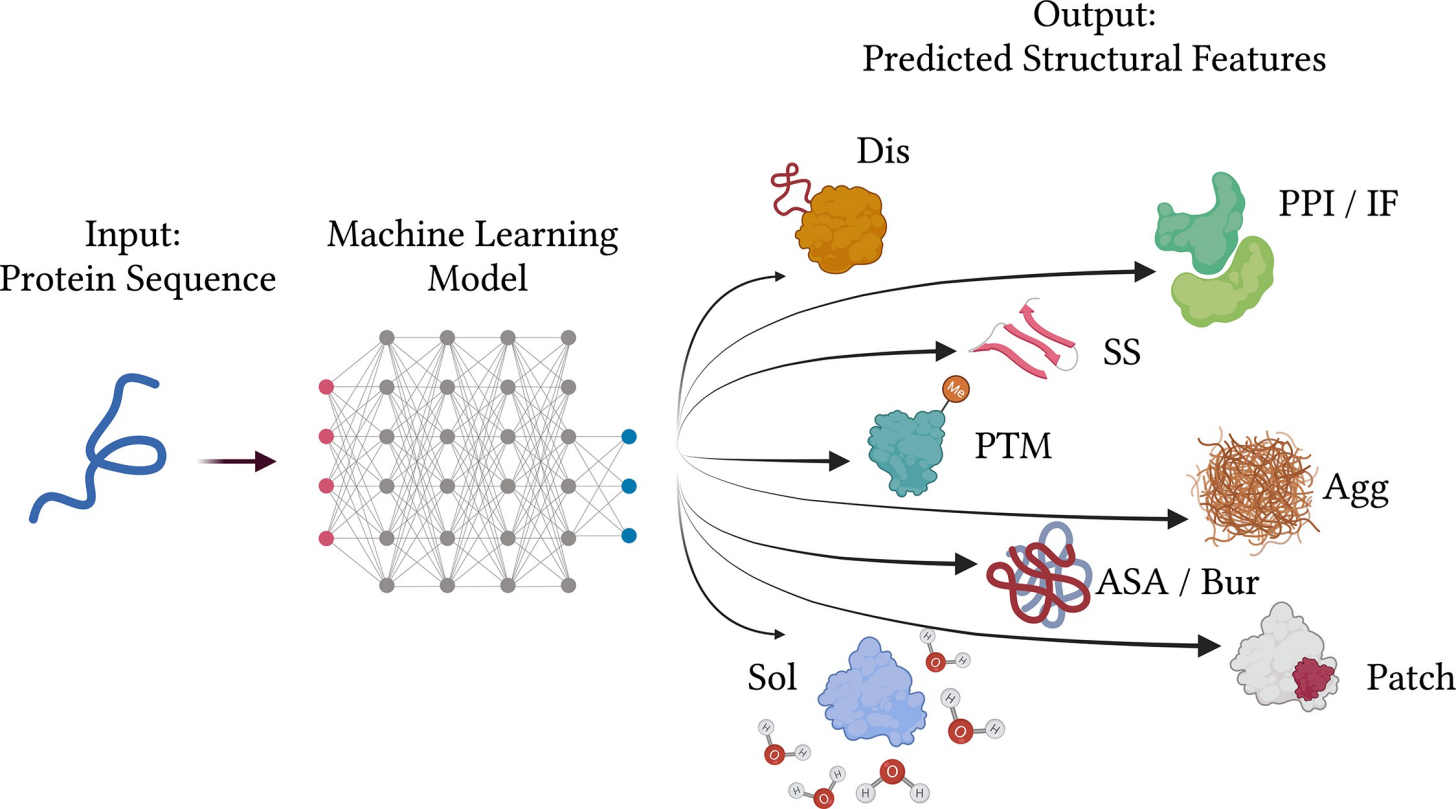
Sequence-based prediction of protein functional structural properties aims to fill the gap between relatively scarce functional annotations, or protein structures, and ubiquitously available sequence data. Predicting structure and function related properties such as disorder *(Dis)*, secondary structure *(SS)*, solvent accessible surface area *(ASA)* or buried vs. exposed residues *(Bur)*, posttranslational modification sites *(PTM)*, large hydrophobic patches *(Patch)*, aggregation propensity *(Agg)*, protein–protein interaction or other interfaces *(PPI/IF)*, or solubility *(Sol)*. Created with Biorender.com.

**Fig 2:**
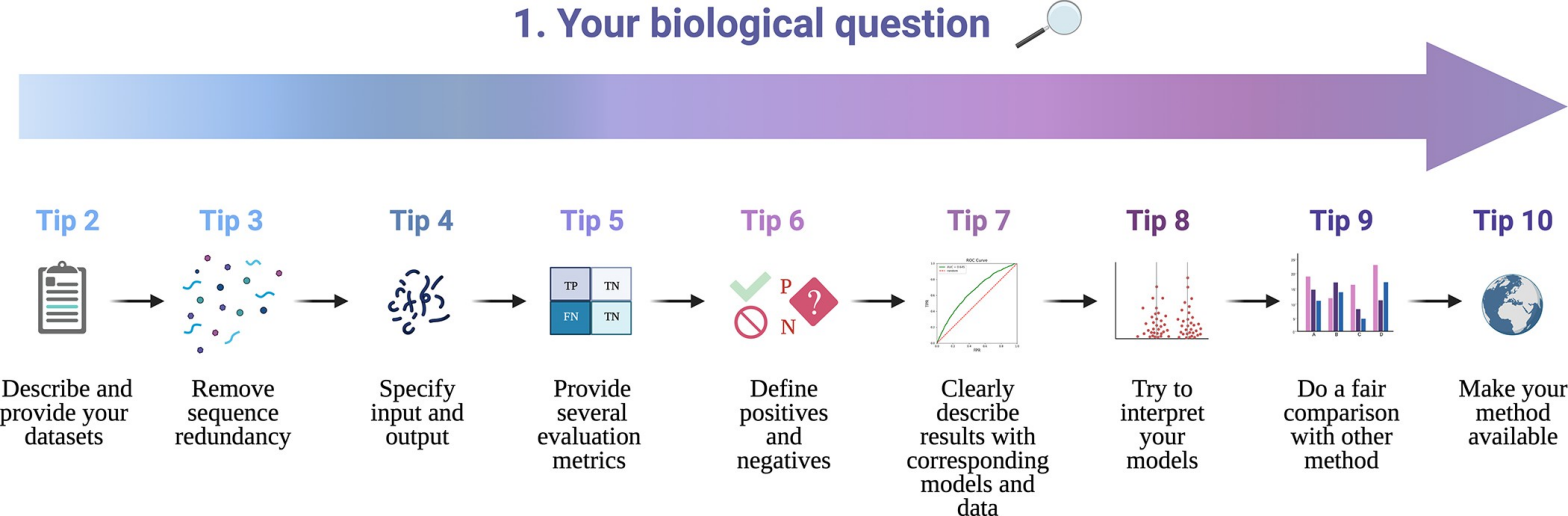
Flowchart of good practices. The main tips on data preparation and benchmark methodology to follow to ensure the usefulness and reproducibility of your work. Created with Biorender.com.

Predicting protein functional properties is still one of the most important tasks for bioinformaticians (e.g., [10–17]). Here, we collect 10 useful tips or guidelines representing best practices specifically for methods that generate *predictions of protein functional structural properties using protein sequence data* as input; Fig 1 illustrates several examples. In Fig 2, we present a flowchart of the proposed tips, starting with the biological question and going through steps of dataset generation, training and testing, and critical comparison and interpretation. We are writing from our own experience in sequence-based prediction of protein–protein interaction (PPI) interfaces [15–19], conformational epitope regions [20], and hydrophobic patches [21]. However, we expect that many of our tips may apply equally well to related tasks, such as prediction of posttranslational modifications or changes of certain properties upon mutation, as well as to structure-based prediction of protein properties.

Structure-based methods generally outperform sequence-based ones. Coupled with the growth of experimentally determined structures, there has been enormous progress in the area of structure prediction in the past several years. In particular, AlphaFold2 constitutes a big leap forward in structure prediction [22]. However, reliable structural information is still not available for many important types of proteins and protein regions (e.g., [23,24]). Moreover, the usefulness of predicted structures as input for prediction of functional properties such as interface regions or binding sites has not yet been validated and may be still quite limited [25–27]. Functional property prediction using structure is not necessarily more accurate than sequence-based approaches (e.g., [26]). Thus, the field of *sequence-based prediction of protein functional structural properties* aims to fill this gap.

Many machine learning model developers in this field have a background either in machine learning and computer science, or in biology, and may take biases and implicit assumptions from their fields, without realising that these may not fit well with the “other” field (e.g., [28,29]). Model developers with a bioinformatics background, who may be expected to have a broader scope, may also fail to realize some of the best practices either in machine learning or protein science. We thus aim to collect tips useful for researchers of different backgrounds working on developing novel prediction models in the protein field. Tip #2 and Tip #5 to Tip #10 are also applicable in general for machine learning approaches; Tip #1, Tip #3, Tip #4, and also Tip #9 are specific for protein-related tasks. Moreover, these tips are applicable to structure-based prediction of protein properties as well.

Following the below instructions will improve the presentation and clarity of your work in your manuscript (and, hopefully, publication). But above all that, following them will help ensure that your research can stand the muster of critical peer review and that your results will be reproducible.

### Tip #1 Answer a biological question

It is easy enough to train a classifier on a dataset. But the more interesting work uses machine learning to answer biological questions. It is important to remember that this is also the first aim of bioinformatics. Your biological question determines the next few points: the dataset to be collected, machine learning methods used, the validation metrics selected, and the methods to compare with. For example, do you want to know which deep learning architecture works best for certain predictions? Or do you simply want to build a novel predictor that is better than any of the available ones? Or are you the first one to develop a method for your particular prediction task? First, you should point out what the biological relevance of that exercise would be. Then, as shown in Fig 2, you should keep your biological question in mind throughout your work.

When introducing your machine learning approach to predicting protein properties from sequence, you want to start from the biological question, and explain why and how your machine learning architecture fits the sequence data used and the biological problem you aim to solve. Protein sequences are not images, nor are they sentences in a language, and if something like syntax or grammar-like structures are applicable, we have only a feeble grasp of how these may work in practice (e.g., [16,17,30,31]). The protein as a whole has some function, a structure to support that, and a long evolutionary background, some of which is reflected in its particular amino acid sequence. This is quite different from images or from sentences in a natural language and would be lost in these analogies. So the challenge is to take the reader from a typical prediction output for protein sequencecs to your machine learning architecture, instead of from cats or English grammar. Thus, while it is appealing to use a broadly known analogy like language to help the reader grasp a concept, including examples from related and well-known biological problems can help the reader understand your work’s relevance and context better.

### Tip #2 Describe and provide your datasets—Training *and* testing

We live in the era of open science and data, and reproducibility of scientific results is of paramount importance (e.g., [3,4,27,32–34]). A good description of your dataset should detail which source databases you used (e.g., the PDB), which selection criteria (e.g., minimum resolution, specific experimental techniques), and which further filtering steps you applied (e.g., on sequence identity, see the next tip). Reproducibility can be improved by providing either the version of the database or the date it was accessed. After data curation, it should be clear how many and which proteins are in your dataset, which sequences you used, and their experimental properties. In machine learning research, it is good practice to also report general statistics on your sequence datasets, which should certainly include class imbalance (covered in more depth by Chicco and colleagues [7]) and amino acid frequencies among others. However, for many biological applications, detailed reporting on the statistics of the datasets used may be over the top. Such type of analysis may fit better when interpreting your model (see Tip #8).

An integral part of your dataset are the features. These may include values directly extracted from the sequences, such as amino acid type, evolutionary information (e.g., profile representations in the form of PSSMs from PSI-BLAST), or features predicted by other tools, such as secondary structure and solvent accessibility. Features may also be learned using representation models such as used in transformers (e.g., [12,31]). These representation models are typically trained on large volumes of unlabeled data and aim to represent the innate structure of the data. There are several pretrained representation models available for proteins (e.g., [12,16,31]). In each case, it should be clear precisely which feature values you took from which tool or source, which scaling if any was applied, and how they are annotated in your datasets—particularly refer to the column headings in your CSV files; we will return to this topic in Tip #8 with concrete examples. Provide a script, or better yet a workflow description in, e.g., the CWL common workflow language [35] (more on this in Tip #10).

More and more journals are already mandating the practice of requiring FAIR sharing of data: Findable, Accessible, Interpretable, Reusable [33]; such as PLoS Computational Biology and Nucleic Acids Research with its annual database issue [36], others are at least encouraging it, such as Bioinformatics, BMC Bioinformatics, Scientific Reports. Greener and colleagues [3] and Zook and colleagues [34] also emphasize this need. Creation of an annotated dataset for protein features is a difficult task, and such a dataset will be valuable for many, provided that it is in a both machine and human readable file format (i.e., not a PDF), self-describing, and well documented (i.e., not just a CSV). Making your data public will also increase the impact of your work, as others may improve on your work without having to re-invent the wheel data and thus cite your paper. Be confident that, when your turn comes round again, you may again improve upon others’ work. This way progress lies!

### Tip #3 Remove sequence redundancy

**Fig 3:**
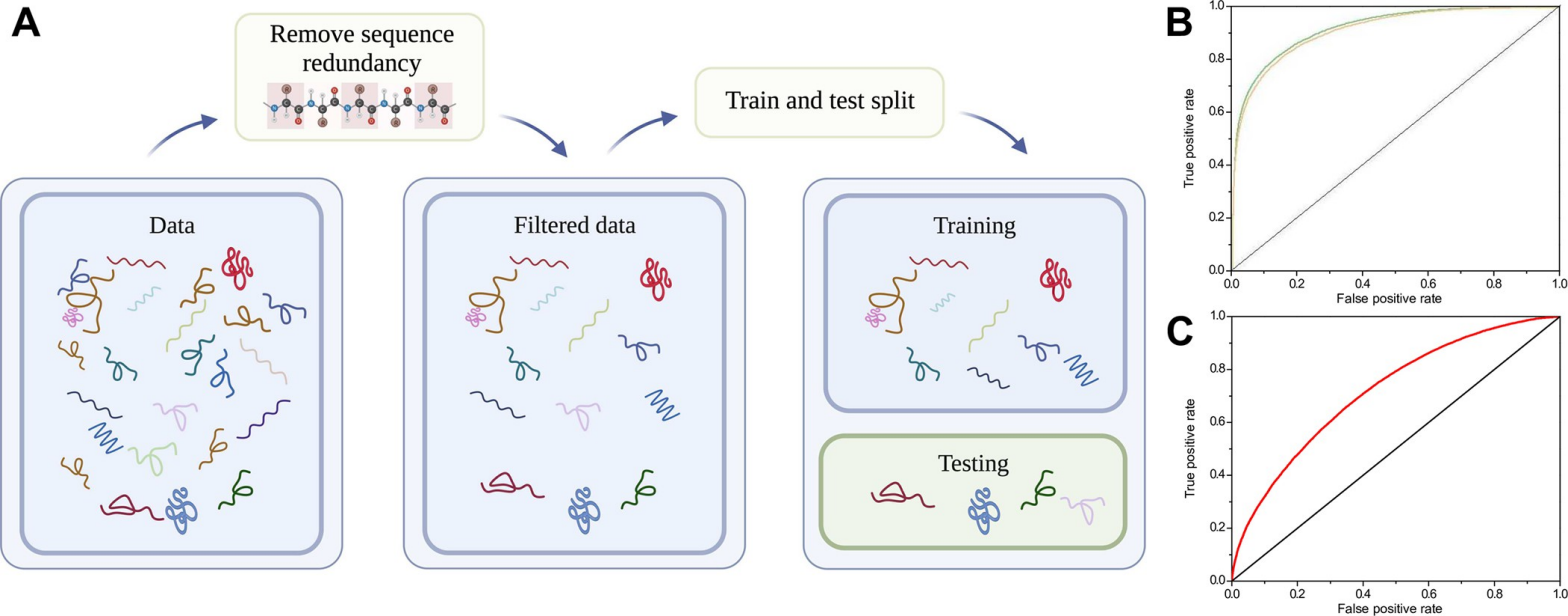
Filter on sequence redundancy. (A) Homologous proteins may end up in different datasets after the train and test split, sharing a large proportion of the amino acid sequence in this case makes the prediction task easy for the machine learning model (created with Biorender.com). (B) ROC plot without redundancy filtering for PPI interface prediction, yielding an unrealistically high AUC of 0.92. (C) In order to avoid this “data leakage” and to make sure that your model is tested and evaluated on data it has not seen yet, your datasets must be filtered on sequence identity before training and testing the model, here yielding an AUC of 0.72. Based on data from Hou and colleagues [37].

If homologs exist between the training and test sets, then your method’s measured performance may be artificially inflated. This is referred to as “data leakage” by Greener and colleagues [3]. By filtering on sequence identity (Fig 3), for example, not allowing any pair of sequences to share more than 25% matching amino acids, you remove sequence redundancy and avoid predicting on homologs between the test set and training set. Of particular note, here is the generation of input features, which was explained in more detail in the previous tip. Typically, you would use existing methods for that. To avoid data leakage by feature-generating methods, sequence redundancy between these methods’ training data and your model’s training data should be removed as well (e.g., [18]).

In deep learning applications, particularly in the pretraining of representation models (e.g., [16]), you would typically like to exploit the full richness of the available data, so it might make sense to build a large *training* dataset that also includes (close) homologs; however, for the *validation* set, it remains vital to minimize data leakage by excluding homologs.

As an alternative to sequence identity filtering, you may exclude proteins from the test set if they are from the same (super)family in CATH [38] or SCOP [39] as proteins in the training set. You may also combine the two approaches, as protein families in CATH and SCOP are particularly tailored to catch remote homologs, which may still exhibit considerable similarity in structure and function. Additionally, it is good practise to check if your training sets may be dominated by one (or a few) large protein families. In such a case, the model may not generalize well for other families. Ultimately, you want your method to work for the whole protein universe and not to be biased towards the most studied protein families.

Finally, there is a trade-off between sufficient data and sequence identity filtering. Sometimes desired cutoffs leave too little data for training a predictor, such as for epitope prediction where cutoffs as high as 50% or 70% antigen protein sequence identity are used (e.g., [40,41]). Therefore, you must find a balance and you should address this in the discussion.

### Tip #4 Specify what the input—and output—of your model is

**Fig 4:**
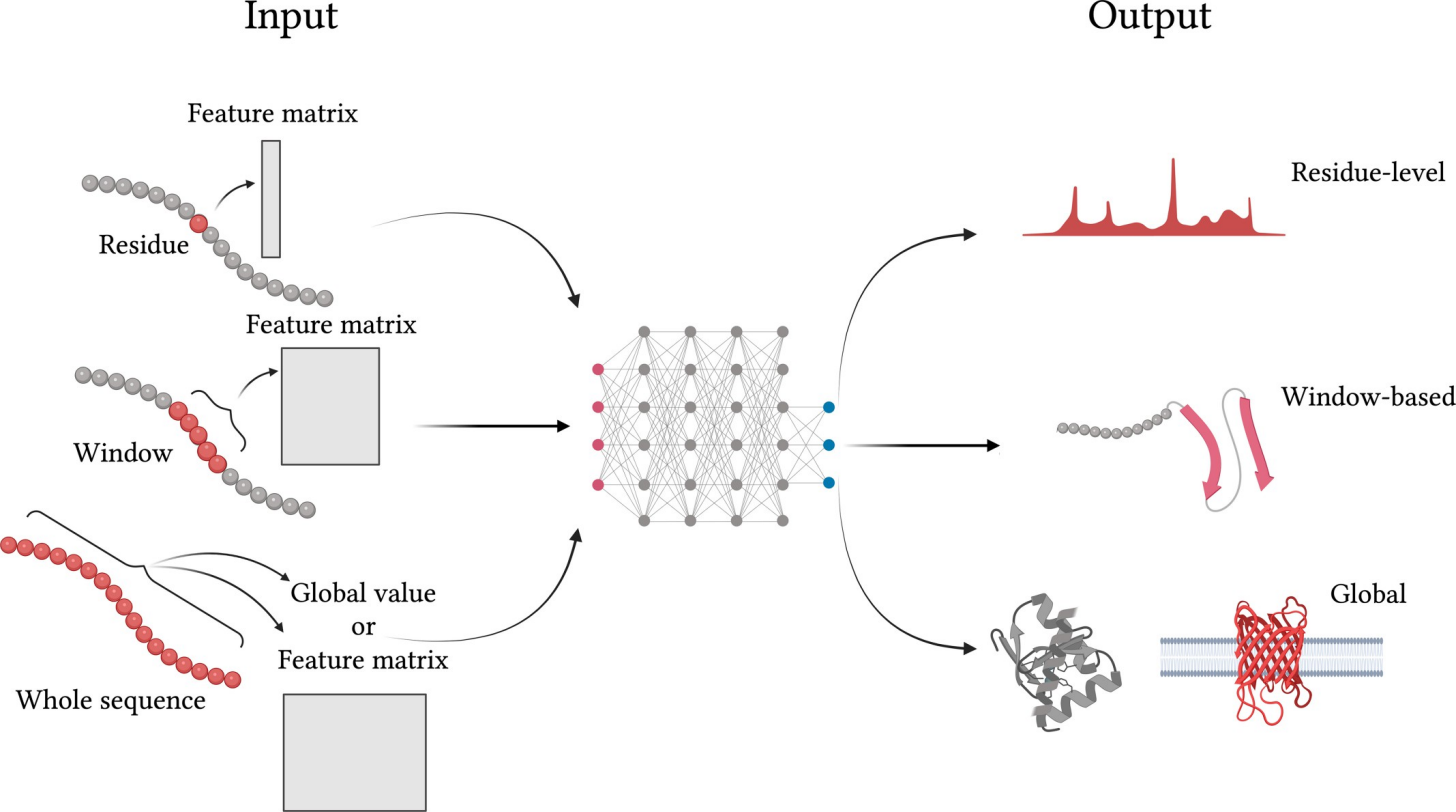
Possible model inputs and outputs. A machine learning architecture may take protein sequence data in different ways: residue-level features, windows or fragments of adjacent residues in the sequence, or a whole protein sequence. Some models may also include global features at the protein level, for example, protein length, amino acid composition, or average hydrophobicity. The output of the model can also vary, including residue-level predictions, region/fragment classification (e.g., secondary structure elements), or protein-level labels (e.g., transmembrane or not). Created with Biorender.com.

We can separate sequence-based methods based on what types of input and output are required, as shown in Fig 4. While the input is always the protein sequence, what is passed to the prediction model may be only a *single residue* at a time, a *sequence window*, or it could be the *whole protein sequence*. In all cases, the actual input to the model are the associated features. Typically, simpler methods like regular neural nets or random forests take only a single residue or window position as input (e.g., [18,20]), while deep learning models obtain their strength from their ability to exploit patterns across the whole sequence (e.g., [15–17]).

The output may be *residue-based*, such as accessible surface area; or *region-specific* properties, e.g., secondary structure elements and disordered regions; or *global protein properties*, such as homology, subcellular location, solubility, and the like. Therefore, you should specify what type of input your model expects and which type of output it predicts, as clearly as possible in the manuscript, and preferably as well in the code itself and the accompanying README file.

### Tip #5 Choose the right benchmark metrics

To successfully introduce your prediction method, and, indeed, to assess it critically, good benchmarking is necessary. One important aspect is selecting the most suitable benchmarking metrics as different metrics highlight different performance properties of your method. Here, we will briefly summarize the most commonly used ones, for their definitions please refer to the Methods section of Hou and colleagueas [20].

Many protein-specific classification tasks have a high class imbalance, for example, generally only about 10% to 15% of the residues in a protein are interacting [17,18]. While being commonly used, *Accuracy* can be misleading when dealing with highly unbalanced data. In this case, the machine learning model simply learns to predict the majority class (non-interacting), while no actual biological patterns are learned from the data. *Balanced accuracy* does not have this problem and is thus preferred. *Matthews correlation coefficient* (MCC) and the *Precision-Recall* (P/R) curve also focus more on the minority class, providing more realistic performance evaluation [7]. The P/R curve may be summarized by the area under the curve (AUC-P/R) or the average precision of the P/R curve (AP). Also, the *ROC curve* (recall versus error) is often used and can be summarized with the area under the ROC curve (AUC-ROC or often simply AUC). The P/R and ROC plots and their AUC are nonparametric, whereas other measures necessitate the rather arbitrary choice of a cutoff point in the predictions. For regression tasks, the most commonly used metrics are *Pearson correlation*, (root) mean square deviation (RMSD), or mean square or absolute error (MSE or MAE).

From a practical point of view, when you wish to compare your method to published ones, you will want to include several overlapping metrics. If you write a benchmarking script (once), it is trivial to calculate all of the above; you may then pick any relevant selection for publication (and put the lot in the supplement).

### Tip #6 Define what a positive—and a negative!—means

None of the measures mentioned in the previous tip make much sense unless you have clearly defined positives and negatives. We have noticed in some cases that it is difficult to extract from a paper exactly how this was done.

**Fig 5:**
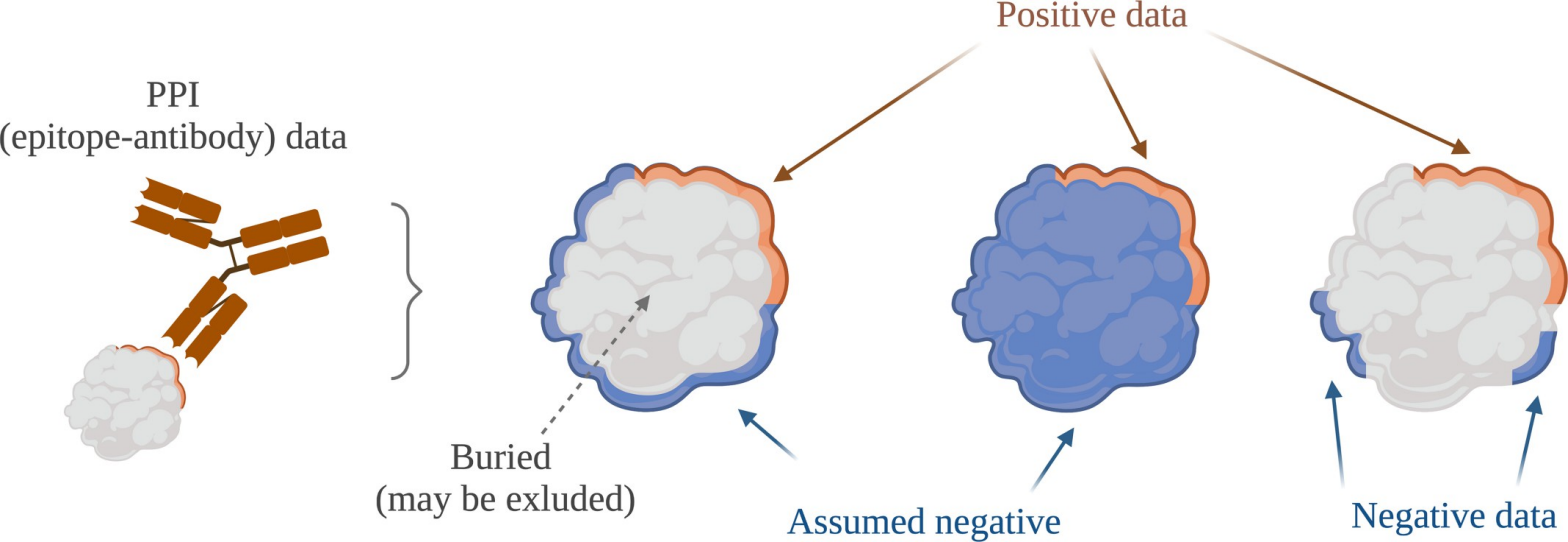
Defining positives and negatives in PPI interface data. A positive is a residue that was observed to be interacting, however, in general it is hard to obtain negative data for PPI. For epitope-antibody binding negative data may be available for some parts of the protein: peptides that were tested and shown to not bind. Buried residues may be considered negatives or you may prefer to exclude them altogether. Created with Biorender.com.

Positives for PPI interface residues or epitope regions may be identified from solved (crystal) structures of bound proteins, as show in Fig 5 (e.g., [17,18,37]). In some cases, a protein may have been crystallized with a binding partner multiple times or even with different binding partners. Were all entries integrated for that protein or was a single one selected? How?

For negatives, things may be even less clear. This is a common problem, as absence of evidence is no evidence for absence. Are negatives simply anything not positive? Were buried residues excluded? Not finding a contact in any crystal structure in the PDB does not mean there is no interaction, rather it could mean that it hasn’t been studied yet. For B-cell epitopes, i.e., epitope-antibody binding that is also a form of PPI, there is some source of negatives as a peptide may have been tested and found not to lead to antibodies being raised; these are real negatives, as also shown in Fig 5 (e.g., [40,41]). But any part of the protein that wasn’t included in any experiment may still actually be a positive.

Hence, you should carefully consider how to define not only positive but also negative entries in your dataset and describe this clearly.

### Tip #7 Clearly describe your results with corresponding models and training/test data

**Fig 6:**
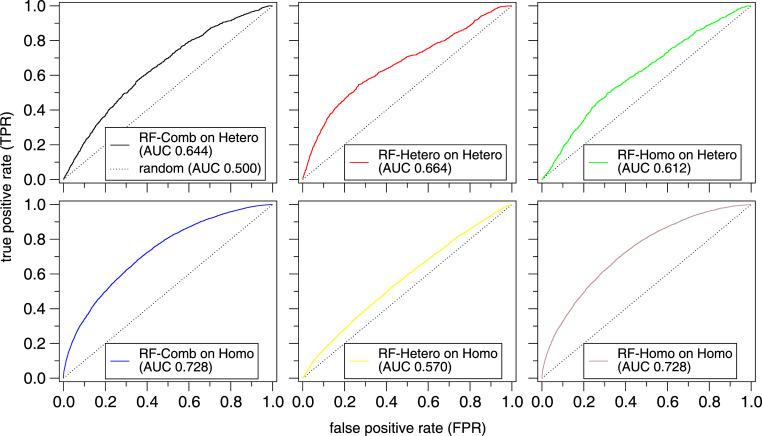
“ROC plots showing the performance of Models RF-Comb, RF-Hetero, and RF-Homo, trained on the combined, heteromeric, and homomeric training sets, respectively, and tested on the heteromeric and homomeric test sets”. When you evaluate several models on different test sets, you should clearly indicate which test set was used for which model followed by the relevant scores. Moreover, if the model names do not readily indicate which training set they were derived from, you should include this in the caption. Created with data from Hou and colleagues [19].

Often, several models, their variations, and corresponding datasets are introduced within a paper. For example, you may include different feature sets, or different architectural building blocks, each leading to a different model. When presenting the results, therefore, it is important to be precise about which model is trained on which training set, which results are based on which model, and which test set. You should use clear and consistent labeling of each of them, for example, by color-coding (e.g., [21]). Simply labeling each of your methods with a short, descriptive name and consistently referring to it by that label throughout the manuscript, avoids a lot of unnecessary confusion (e.g., [17]). The same applies to datasets. Often, published datasets are already named, otherwise name it, e.g., by author initials and size of the dataset (number of proteins), like the 448-protein test set “ZK448” for protein interactions by Zhang and Kurgan [42]. We give a simple example for how this might be done in Fig 6.

### Tip #8 Try to do an intelligent interpretation of your model, at least of feature importance

**Fig 7:**
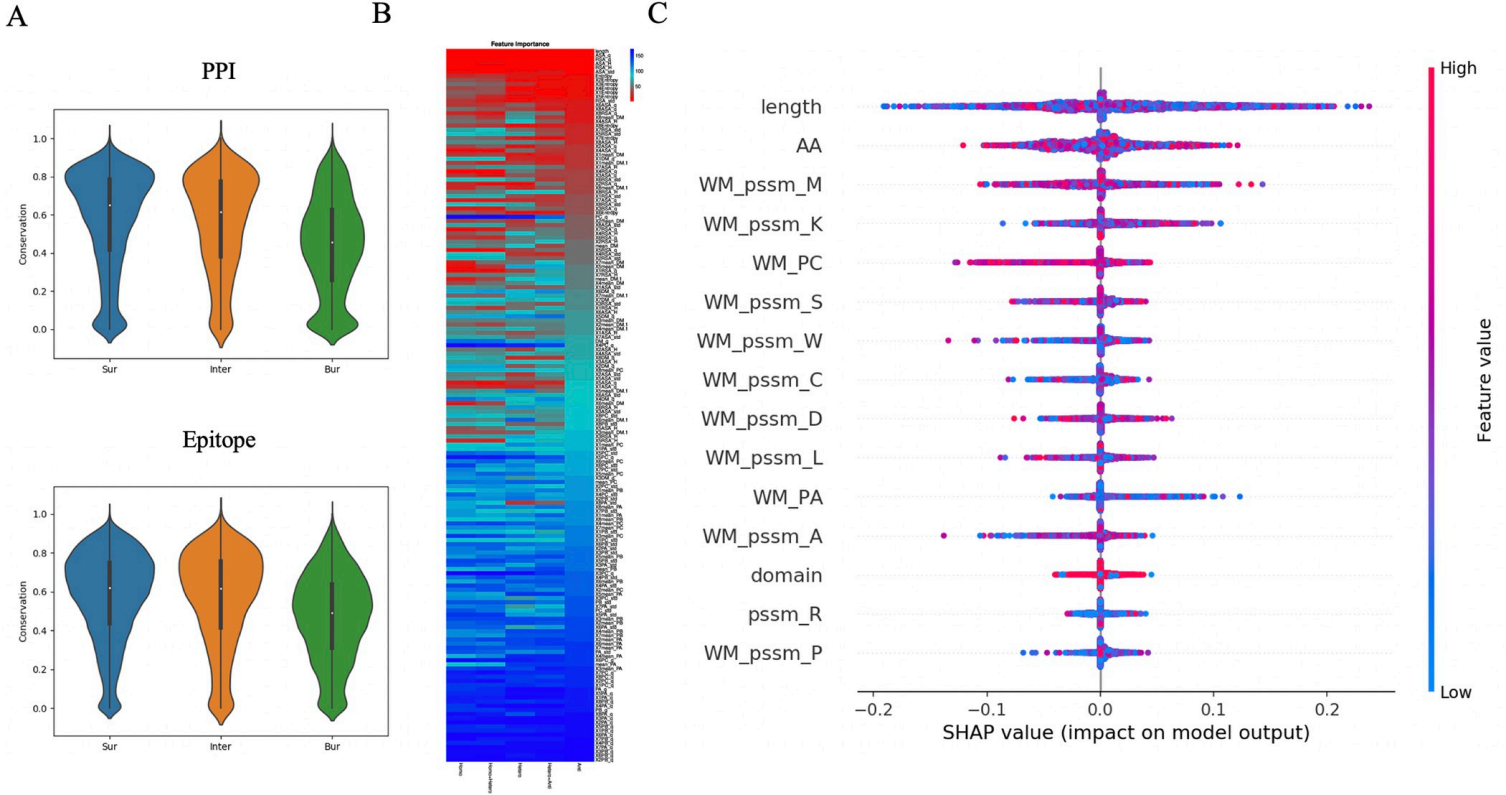
Various ways to interpret your model. (A) Simply checking (cor)relation between class labels and certain features of interest; the example shows how the pattern of conservation differs between surface (Sur), interface (Inter), and buried (Bur) residues for protein–protein (top, PPI) and epitope interactions, which are a specific type of PPI (bottom); based on data from Hou and colleagues [37]. (B) GINI feature importance, which is a simple measure per feature indicating how much each contributes to the predictions, here shown as a heat-plot (features as rows) for 5 different models for PPI and epitope prediction (columns) [20]; one may appreciate how some features are prominent across models (bright red across), while others appear to be more model specific (only red in some columns). (C) SHAP plot, which is an additive score that estimates per datapoint (single points) and per feature (along vertical axis) what the effect of the feature value (color) is on the prediction (horizontal axis) [17]; most features can be seen to have many strong effects, both positive and negative. The top feature is sequence length, then amino acid type (AA), and the other features are related to the PSSM profile per amino acid type and propensities for coil (PC) or helix (PA); for most a window mean (WM) is taken.

Some machine learning methods, and especially deep learning approaches, are notorious for their black-box nature. A trade-off between accuracy and interpretability leads to the best-performing models often providing the least amount of insights into their decision-making. The arising interpretation difficulties become paramount particularly in medical applications. We have sometimes seen authors take an off-the-shelf machine learning tool, apply it on their dataset, and take the results for granted. Leaving matters at the “black box” level, however, is a huge opportunity lost, and these models can be better interpreted [3,4], but sometimes you need an expert to help [6]. Many methods have been developed to at least let some light out of (or into?) the box, so that at least some of the main characteristics of the decision-making process may become clear. Some examples are shown in Fig 7. A straightforward analysis, often performed before the actual machine learning, is to inspect differences in feature values between labels, shown in Fig 7A. For random forest models, the “GINI impurity” is regularly used to rank features based on their importance inside the decision tree models, shown in Fig 7B (eg., [18,20]). It is also possible to look at “feature interactions,” that will help you find which features are very often used together in decision rules. For neural net models, particularly the more complex ones, the SHAP analysis provides additive scores of feature importance, shown in Fig 7C (e.g., [17,43]). As mentioned in Tip #2, clearly describing and labeling your features is crucial, as understanding your interpretation figure—which inevitably uses abbreviations—becomes impossible for the reader, as can be readily appreciated from Fig 7B and 7C.

A good way to validate the impact of a particular feature is to remove it from the input, then retrain, and test again. We have done this with the global feature of protein length showing that AUC-ROC values drop considerably, significantly (see the next tip), and consistently [17–20]. You may also want to do it the other way around, train on only some of the most important features, to show how much (or how little) the other features still add (e.g., [18]).

As the retraining may be fairly computationally costly, and you may wish to evaluate multiple features, and even many combination of features, a cheaper method is zero-ing out the features in the input of the model. (This is also known as “ablation” in machine learning, but note that there are other types of ablation, notably by removing components from, e.g., a neural net model.) However, this may prevent the model from being able to compensate for the missing information by employing similar information that may be obtained from other features or combinations thereof.

### Tip #9 Do a fair comparison with other methods

Benchmarking in bioinformatics includes comparison with related, preferably published, methods (e.g., [3,4]). Firstly, there isn’t always a clear reason for a head-on comparison with other methods. You may, for example, be setting out to find the added value (or not) of specific parts of your training procedure (e.g., [15]) or of the architecture (e.g., [16,17]). First point of business will be to identify the current state of the art, which you can usually find in a recent benchmarking review. For PPI prediction, an excellent example is Zhang and Kurgan [42], who also published their ZK448 test set. To this comparison, we recently added several neural net architecture models, and a large data set BioDL for PPI, nucleic acid, and small molecule interactions [17]. We also introduced a broader benchmark set ProteinGLUE including mutiple prediction tasks: secondary structure, solvent accessibility, PPI, epitopes, and hydrophobic patch prediction [16]. Many method papers will also include an update of latest developments (e.g., [15,44]).

If you state that you compare to “state-of-the-art,” be certain that you have the two or three best scoring methods (and reference a recent review, preferably not your own). To make sure that your comparison with other methods is fair, you have to “level the playing field” as much as possible. Make sure that the definitions of positives and negatives are identical (or fairly similar). You should only compare metrics for methods tested on (exactly) the same test sets; or if that is the only viable comparison, at least clearly indicate which test set was used where, and what the important differences between the test datasets are. As discussed in Tip #2 and Tip #3, assess the overlap in sequence similarity between (all) test set(s) used, and all relevant training sets including those of methods that were used to generate the input features. For maximum fairness, also test *your* method on *their* dataset, not only their method on your dataset. By doing this, you will also be able to assess better how your method may perform on different datasets.

An issue we encountered several times is the claim of methods to be sequence-based that simply aren’t: they sneak in structural data by using structure-derived secondary structure annotations from the DSSP database as input features. For interface prediction, we have seen this happen in a number of published papers. This becomes important when benchmarking against actual *sequence-based* methods, as these methods cannot benefit from the advantage of structural information. While outperforming other tools in a direct comparison, these methods do not promote the ultimate goal to predict protein properties when no structure is available.

As the main part of benchmarking is comparison, it makes sense to attach a significance estimate to each difference that you would like to conclude is important. Based on your significance estimates, you may also want to reduce the number of digits you report, e.g., in tables. A straightforward way to estimate significance is by cross-validation, i.e., split your training set into 5 parts, train a model on each of those, and estimate a *p*-value of the observed difference in performance. For AUC-ROC scores, a direct *p*-value estimation can be done online [45].

Typically, any differences of 1 percent or smaller are either insignificant or uninteresting. For most metrics, this means 2 decimal places after the decimal point (or only whole numbers if you express them as a percentage); including more digits will only make your results less readable.

### Tip #10 Make your method available

Many journals, including PLOS Computational Biology, Bioinformatics, and BMC Bioinformatics, already require the method to be published with the paper, either as code or as a web server. For others researchers to be able to use and compare your method, or continue on your work, it is essential that your method is available. Obviously, your paper includes a detailed description of how it was done, but also publicly available code and a working web-server are important. Re-creating a method from the paper is tedious and often practically impossible. Even running someone else’s code can prove to be very difficult, and in addition many potential users of your method may not have the tech-savyness required for overcoming these difficulties. A container solution, for example, Docker or Singularity, resolves some of these problems such as tracking tool versions, but adds its own complexity [46,47]. Generalized workflow descriptions, such as in the Common Workflow Language (CWL) [35] or Galaxy [48,49], aim specifically to improve portability of developed methods, including tracking of software versions, and are thus well suited for sharing methods. Other initiatives aim to simplify the sharing of (trained) machine learning models, such as OpenML [50] or ONXX [51], and there are ongoing efforts to improve reproducibility and assessment in general in machine learning applications (e.g., [9]). Finally, for many of your users a web-server will be well appreciated the most; many of these are collected in the annual web-server issue of Nucleic Acids Research (e.g., [49,52,53]), but may also be found, for example, as Software in BMC Bioinformatics (e.g., [54]) or Application Note in Bioinformatics (e.g., [19]).

In addition to reporting on the run times of your prediction method (listed in CPU or GPU hours), it is also important for the users to know the computational cost of generating the input features and predictions. Especially when testing your method on their own dataset, users would like to know if the prediction takes seconds, minutes, or perhaps even hours per protein.

## Discussion and conclusion

In this paper, we collected 10 important tips that you, as sequence-based functional structural properties prediction tool developer, should pay attention to during the processes, starting from raising the biology question, preparing the datasets to training and testing, all the way to making your predictor available to the community. Several tips could be also applied—even quite obviously—to the more general machine learning field, and also to methods that use protein structure features as input. However, from our experiences with manuscripts and published papers, each of our tips has been neglected at least once in this field.

There are many issues that we did not discuss here. We have not gone into many machine learning-specific points, such as ensuring that your method is a good match with the type and amount of available data and with your prediction target of choice, including assessing whether your prediction problem falls under clustering, classification, segmentation, or regression. Also, issues of overfitting were not covered explicitly, although they are related to Tip #3: proper filtering on sequence identity. Still, there are many other strategies to reduce this risk. To learn about these additional issues, and more, we refer to the excellent summaries about matching biological question with algorithm, and proper model training by Chicco [7] and Lee and colleagues [4], more background on machine learning (“AI”) in general by Malik and colleagues [6], and choosing and assessing the appropriate datasets, models and results by Greener and colleagues [3] and Lee and colleagues [4]. A different aspect of big data in biology, and particularly biomedicine, is that it is intricately privacy sensitive, as discussed in detail by Zook and colleagues [34], and also addressed by Malik and colleagues [6] and Greener and colleagues [3]. In addition, Lee and colleagues [4] provide an overview focused on deep learning approaches discussing several crucial aspects, namely choosing appropriate models for your problem, performance baselines, complexities of model training, and more.

Future machine learning models will likely be increasingly complex, with a growing need for better interpretability and more rigorous testing. It is also important to realize the inherent limitations of the technology, as has been pointed out by many others (e.g., [3,6]). Often, artificial intelligence makes mistakes and to us, humans, it may not at all be clear why. For science, as humanity’s vehicle to better the understanding of our world, this is an unacceptable situation, but there doesn’t seem to be a ready-made solution to this, as of yet.

Despite the many pitfalls, machine learning approaches have much to offer in the field of protein structural property prediction and are here to stay. With structural data also becoming increasingly common [55], more and more approaches will be able to benefit, and the tips presented here will continue to be useful also for structure-based approaches. Methodology improves, databases grow, and we see the scientific community sometimes struggling to keep up. New methods should be tested rigorously but fairly on their merits, whether they aim to provide proof of principle for some innovation, or whether the goal is to provide the most accurate predictions, or some better compromise between cost (speed) and accuracy. Best practices for publication should continue to keep pace with these developments, as should the researchers. It is our hope and expectation that the current overview provides them with a good place to start.
